# Altered Functional Brain Connectomes between Sporadic and Familial Parkinson's Patients

**DOI:** 10.3389/fnana.2017.00099

**Published:** 2017-11-06

**Authors:** Yan Tang, Xue Xiao, Hua Xie, Chang-min Wan, Li Meng, Zhen-hua Liu, Wei-hua Liao, Bei-sha Tang, Ji-feng Guo

**Affiliations:** ^1^Department of Neurology, Xiangya Hospital, Central South University, Changsha, China; ^2^School of Information Science and Engineering, Central South University, Changsha, China; ^3^School of Basic Medical Science, Central South University, Changsha, China; ^4^Department of Electrical and Computer Engineering, Texas Tech University, Lubbock, TX, United States; ^5^Department of Radiology, Xiangya Hospital, Central South University, Changsha, China; ^6^Parkinson's Disease Center of Beijing Institute for Brain Disorders, Beijing, China; ^7^Collaborative Innovation Center for Brain Science, Shanghai, China; ^8^State Key Laboratory of Medical Genetics, Changsha, China

**Keywords:** functional brain connectome, sporadic PD, familial PD, assortativity, small-worldness

## Abstract

Familial Parkinson's disease (PD) is often caused by mutation of a certain gene, while sporadic PD is associated with variants of genes which can influence the susceptibility to PD. The goal of this study was to investigate the difference between the two forms of PD in terms of brain abnormalities using resting-state functional MRI and graph theory. Thirty-one familial PD patients and 36 sporadic PD patients underwent resting-state functional MRI scanning. Frequency-dependent functional connectivity was calculated for each subject using wavelet-based correlations of BOLD signal over 246 brain regions from Brainnetome Atlas. Graph theoretical analysis was then performed to analyze the topology of the functional network, and functional connectome differences were identified with a network-based statistical approach. Our results revealed a frequency-specific (0.016 and 0.031 Hz) connectome difference between familial and sporadic forms of PD, as indicated by an increase in assortativity and decrease in the nodal strength in the left medial amygdala of the familial PD group. In addition, the familial PD patients also showed a distinctive functional network between the left medial amygdala and regions related to retrieval of motion information. The present study indicates that the medial amygdala might be most vulnerable to both sporadic and familial PD. Our findings provide some new insights into disrupted resting-state functional connectomes between sporadic PD and familial PD.

## Introduction

Parkinson's disease (PD) is a neurodegenerative disease with symptoms such as resting tremor, rigidity, akinesia and postural instability as shown by Parkinson (2002). It was reported by Hauser and Hastings (2013) that in about 95% of PD cases, there is no apparent genetic linkage (sporadic PD), while for the rest cases, the disease is inherited and have monogenic forms (familial PD). Familial PD is often caused by mutation of a certain gene, while sporadic PD is associated with variants of genes which can influence the susceptibility of developing PD (Guo et al., 2008; Ciceri et al., 2017). Thompson et al. (2001) has reported that genes affecting the development and function of brain that mediated the expression of such diverse behavioral, cognitive and perceptual phenomena. Dujardin et al. (2001) provided evidence that sporadic PD and familial PD differ in terms of cognitive impairment that deficits of explicit memory recall were only observed in patients with sporadic PD. Despite the recent discovery of the mutation patterns, the two forms of PD are difficult to distinguish clinically and pathologically (Chai and Lim, 2013), and the underlying disease-related neural mechanism of different forms of PD remains elusive. As suggested by Palop et al. (2006) that neurodegenerative diseases may lead to network dysfunction, we decide to explore the brain's potential functional network difference between sporadic PD and familial PD to further our understanding of the pathogenic mechanism difference between the two.

Resting functional Magnetic Resonance Imaging (R-fMRI) measures the intrinsic or spontaneous neuronal activity of the brain (Fox and Raichle, 2007) and has shown PD-related breakdowns in functional brain synchronization (Hacker et al., 2012; Tessitore et al., 2012). Recent developments in the quantitative analysis of complex networks using graph theory, have enabled the exploration of the network architectures of brain system (Sporns et al., 2005). Moreover, many studies have suggested the potential link between various brain diseases and altered topological patterns in the functional connectivity (Lynall et al., 2010; Rubinov and Sporns, 2010; Sanz-Arigita et al., 2010). Graph theory has also been applied to investigate the abnormal functional brain network in PD patients. For example, Göttlich et al. (2013) and Fang et al. (2017) both found PD exhibited disruptive visual networks in early disease stage; Luo et al. (2015) found the decreases in local efficiency and local clustering coefficient in the weighted brain network of drug-naive PD patients. The above-mentioned studies have shed some light into the abnormal connectome associated with PD but the differences in terms of the topological architectures of brain connectome present in sporadic PD and familial PD remain largely unknown.

The BOLD power spectrum exhibits scale-free feature. As suggested by Baria et al. (2011), the lower frequency band exhibits the higher magnitude including prefrontal, parietal, and occipital cortices, especially within several default-mode regions; the higher frequency band exhibits less power, and localizes more within subcortical structures (e.g., thalamus and basal ganglia). Independent frequency bands are generated by distinct oscillators with specific properties and physiological functions highlighting the significance of frequency information in neural oscillations (Buzsáki and Draguhn, 2004; Zuo et al., 2010). In fact, the frequency-dependent intrinsic activity pattern altered by diseases have been widely reported, e.g., Alzheimer's disease (Li et al., 2017), PD (Zhang et al. (2015), and schizophrenia (Yu et al., 2014). More recently, the wavelet-based frequency analysis (Percival and Walden, 2006) has been applied in conjunction with resting-state functional connectivity (RSFC) to further the understanding of the pathophysiologies of various diseases including mild cognitive impairment (Wang et al., 2013), antisocial personality disorder (Tang et al., 2016), and Alzheimer's Disease (Supekar et al., 2008). Skidmore et al. (2011) and Zhang et al. (2015) both discovered that in PD patients, the information translation in the functional brain network was disrupted within the wavelet scale 2 (i.e., 0.063–0.125 Hz), which could be related to acute dopamine depletion. However, Dujardin et al. (2001) emphasized the contribution of nondopaminergic loops to development of sporadic PD. Along those lines, we followed an earlier study investigating PD-related spontaneous brain activity (Zhang et al., 2015) and decomposed RSFC into four wavelet scales (scale 1, 0.125–0.250 Hz; scale 2, 0.063–0.125 Hz; scale 3, 0.031–0.063 Hz; and scale 4, 0.016–0.031 Hz). We aimed to investigate frequency-dependent difference in the global and local functional network topology alternation between sporadic and familial PD.

## Materials and methods

### Ethics statement

This study was performed in accordance with the recommendations of the ethics committee of Central South University (Changsha, China) with written informed consent from all subjects. All subjects gave written informed consent in accordance with the Declaration of Helsinki. The protocol was approved by the ethics committee of Central South University (Changsha, China).

### Subjects

The PD patients were diagnosed based on their history and a neurologic examination conducted by a fellowship-trained movement disorders specialist. The clinical diagnosis of PD was made based on the UK Parkinson's Disease Society Brain Bank (UKPDBB) criteria (Hughes et al., 1992, 2001) by two or more experienced neurologists. Each patient with definite PD and accompanying family members were interviewed for Parkinson's disease, Parkinson's disease-like symptoms and any other central nervous system (CNS) disorder among the first or second degree relatives. When a secondary case of PD was found in a family, this affected person was examined by a neurologist based on UKPDBB criteria to make a definitive diagnosis of Parkinson's disease. If no other occurrence of PD was identified in the family after thorough investigation, the case was considered as sporadic. Relatives reported to be unaffected by PD or any other CNS disorder were not examined, as Maraganore and colleagues found no false negatives among relatives reported as normal (Maraganore et al., 1991).

Sixty-seven PD patients were recruited for the study. The patients consisted of 31 familial PD patients with at least one relative of the first or the second degree also identified as PD, according to the criteria previously described. The exclusion criteria included moderate-to-severe head tremor, other neurological diseases (e.g., severe head trauma or stroke), and other general exclusion criteria for MRI scanning (e.g., claustrophobia, wearing pace-maker, and implanted metal parts).

All patients were studied in the off-medication state after all antiparkinsonian drugs had been withheld. Before the patients were scanned, they underwent a series of neurological examinations and neuropsychological testing including assessments with the Unified Parkinson's Disease Rating Scale (UPDRS) (Fahn and Elton, 1987), the Hoehn and Yahr (H-Y) stage (Hoehn and Yahr, 1967; Goetz, 2003), the Mini-Mental Status (MMSE) (Folstein et al., 1975) and Hamilton Depression Scale (HAMD) (Hamilton, 1960).

### Image acquisition

All scans were performed using a 3T GE Signa MR scanner (General Electric, Fairfield, CT, USA) in the Department of Radiology of Xiangya Hospital of Central South University. Earplugs were used to reduce scanner noise, and head motion was restricted via a foam pillow and extendable padded head clamps. The participants were instructed to rest with their eyes closed, relax and lie still. Functional scans were acquired using a gradient echo EPI (GE-EPI) sequence with the following parameters: *TR* = 2,000 ms, *TE* = 30 ms, slice thickness = 4.0 mm, flip angle = 90°, slice number = 32 and voxel size = 3.44 × 3.44 × 4.60 mm. One hundred eighty image volumes were acquired for each participant over the course of 6 min.

### Data preprocessing

Data preprocessing was carried out using SPM8 (www.fil.ion.ucl.ac.uk/spm); and DPARSF (Chao-Gan and Yu-Feng, 2010) (http://www.restfmri.net). The first 10 volumes were discarded to achieve scanner equilibrium. The remaining 170 images underwent slice timing and motion correction, and no subjects were found with head motion more than 1.5 mm of translation or greater than 1.5° of rotation in any of the x, y, or z axis. Next, the images were spatially normalized to Montreal Neurological Institute (MNI) space with the voxel size of 3 × 3 × 3 mm. No spatial smoothing was performed to avoid degradation of diagnosticity (Kriegeskorte et al., 2006). The resulting volumes were detrended and temporally filtered using a high-pass filter (cut-off frequency = 0.01 Hz). Finally, the nuisance signal regression was performed with six rigid-body motion parameters, white matter and cerebrospinal fluid signal (Desjardins et al., 2001; Fox et al., 2009). Because of the controversy of global signal regression (GSR) during the preprocessing which may create anti-correlated patterns (Fox et al., 2009; Murphy et al., 2009), GSR was not performed following previous studies of wavelet-based functional brain networks (Di Martino et al., 2009; Lynall et al., 2010; Wang et al., 2013). Functional connectivity was then computed on the residual signal using wavelet-based frequency analysis.

### Network construction

The processed images were parcellated into 246 regions of interests (ROIs) that included 210 cortical and 36 subcortical subregions according to the Brainnetome atlas (Fan et al., 2016). Brainnetome atlas is a fine-grained, cross-validated atlas correlating brain anatomy with psychological and cognitive functions. In the following graphical analysis, each ROI represented a node in the functional brain network. The mean time series were computed by averaging the BOLD signal of all voxels within each ROI.

Wavelet-based frequency analysis has been brought up to further the understanding of the pathophysiologies of various diseases (Percival and Walden, 2006; Tang et al., 2016). In order to measure the intrinsic pattern of specific frequency bands, discrete wavelet transform was applied to compute the interregional frequency-dependent RSFC, which reflects the functional associations between brain regions evoked by activities of different frequency intervals and wavelet scales (Maxim et al., 2005; Achard et al., 2006). Here, functional connectivity was estimated in the first four wavelet scales (scale 1, 0.125–0.250 Hz; scale 2, 0.063–0.125 Hz; scale 3, 0.031–0.063 Hz; and scale 4, 0.016–0.031 Hz) as in Zhang et al. (2015). The correlation matrices were then converted into adjacency matrices A_ij_ = [a_ij_]. To control a family-wise significance level of 0.05 of 0.05, Bonferroni adjusted *p*-value threshold was computed as 0.05 divided by the total number of links (246 × 245/2 = 30,135) = 1.659 × 10^−6^. The entry a_ij_ was the Pearson's correlation coefficient if the Bonferroni adjusted *p*-value was below the statistical threshold (*p* < 0.05, Bonferroni-corrected) and was set to 0 otherwise, as shown in Equation (1).

(1) aij={Pearson's correlationif p<0.05,Bonferroni−correctedcoefficient 0otherwise 

Considering the ambiguous physiological meaning of negative correlations, negative connections were also set to zero.

### Network analysis

To characterize the topological organizations of weighted functional networks, the following graph theoretical measures were evaluated: clustering coefficient, characteristic path length, small-worldness, assortativity, and nodal strength. The clustering coefficient (C_p_) was defined as the average of the clustering coefficients over all nodes reflecting the possibility that the neighbors of a vertex would also be connected. C_p_ is between the range of 0 and 1, and is equal to 1 if and only if the network is fully connected. The characteristic path length L_p_ is the average of the length of the shortest path between the all pairs of vertices, and it is a measure of the overall routing efficiency of the network. To assess the small-worldness of the constructed functional networks, we simulated 1,000 random networks via random rewiring using the same number of nodes, mean degree and degree distribution of the real network, and calculated the mean clustering coefficient ${\text{C}}_{\text{p}}^{\text{rand}}$, and the mean characteristic path length ${\text{L}}_{\text{p}}^{\text{rand}}$ of the 1,000 simulated random networks. A network with small-world topology can be characterized as follows: $\gamma =\frac{\text{Cp}}{{\text{C}}_{\text{p}}^{\text{rand}}}>1,\;\lambda =\frac{\text{Lp}}{{\text{L}}_{\text{p}}^{\text{rand}}}\approx 1\text{ and }\sigma =\frac{\gamma }{\lambda }>1$.

The resilience of brain systems could be impaired by various diseases. To assess the resilience of the brain network, the assortativities of four wavelet scales were computed, using Equation (2) (Newman, 2002; Achard et al., 2006):

(2) $$\text{r}=\frac{\frac{1}{\text{T}}\sum_{\text{j}>\text{i}}{\text{k}}_{\text{i}}{\text{k}}_{\text{j}}{\text{a}}_{\text{ij}}}{\frac{1}{\text{T}}\sum_{\text{j}>\text{i}}\left({\text{k}}_{\text{i}}^{2}+{\text{k}}_{\text{j}}^{2}\right){\text{a}}_{\text{ij}}-{\left[\frac{1}{\text{T}}\sum_{\text{j}>\text{i}}\frac{1}{2}\left({\text{k}}_{\text{i}}+{\text{k}}_{\text{j}}\right){\text{a}}_{\text{ij}}\right]}^{2}}$$

where *T* is the number of the links in the network, *a*_*ij*_ is the corresponding element of the adjacency matrix between nodes *i* and *j*, and *k*_*i*_ is the degree of node *i*. In general, *r* lies between −1 and 1. An assortative network has positive assortativity, which indicates a network with more mutually coupled hub nodes, whereas *r* < 0 implies a disassortative network in where high-degree hub nodes are unlikely to be inter-linked (Barzegaran et al., 2012).

Although many nodal metrics could be used to characterize regional centrality (Zuo et al., 2012), we employed the nodal strength (i.e., the weighted degree centrality) because of its high test-retest reliability (Wang et al., 2011). The nodal strength of a given node in a network is defined in Equation (3) as the sum of all edge weights a_ij_ between a given node and all other nodes in the network:

(3) $${\text{k}}_{\text{i}}=\sum_{\text{j}\in \text{N}}{\text{a}}_{\text{ij}}$$

### Statistical analysis

Group differences in the topological architectures (global measures) were characterized using nonparametric permutation tests. For each network metric, we first calculated the between-group difference in terms of the mean network metrics. Then, we randomly reallocated all values into two groups and recomputed the mean differences between the two randomized groups. The permutation was repeated 10,000 times, and the 95th percentiles of the empirical distribution were used as the critical values for a two-tailed *t*-test of the null hypothesis with a probability of type I error of 0.05.

Regarding the multiple exploratory analyses of the nodal measures, the multiple comparisons correction was performed for nodal strength (*p* < 0.05, FDR-corrected).

The difference of RSFC between the familial and the sporadic groups were determined utilizing a network-based statistical (NBS https://sites.google.com/site/bctnet/comparison/nbs) approach. A primary threshold was applied to keep a subset of supra-threshold links. Then a corrected *p*-value was computed using the null distribution of the maximal connected component size, which was empirically derived from 10,000 nonparametric permutations. Further details of the NBS approach can be found in the prior work (Zalesky et al., 2010).

### Reproducibility

The reproducibility of our results was addressed in terms of treatment of negative correlations, motion effect and comparison against health controls.

First, considering significant negative correlations found in many diseases, negative correlations were kept to analyze the functional connectomes including global measures and nodal measures.

Secondly, although no significant group difference was found between the familial and sporadic PD patients (*p* > 0.05) in terms of motion, the “scrubbing” method was used to substitute the frames with frame-wise displacement over 0.5 mm by linearly interpolating neighboring frames (Power et al., 2012). Then, the resulted “scrubbed” data were reanalyzed.

Thirdly, in order to investigate whether these abnormal functional connectomes are only present between the familial and sporadic PD patients, the difference of RSFC between PD patients and healthy controls were also studied (see Supplementary Material). A group of 38 age- and sex-matched healthy controls was analyzed, who showed no parkinsonian symptoms nor received any neuroleptic treatment.

## Results

### Demographic and psychometric information

Demographic information, duration of disease, disease stage, UPDRS score and medications are summarized in Table 1. As shown in Table 1, there were no age or sex differences between two PD groups (two-sample *t*-test p_age_ = 0.899; Chi-square test p_sex_ = 0.745), and no between-group differences were found in the duration of disease (two-sample *t*-test, *p* = 0.411), MMSE (two-sample *t*-test, *p* = 0.063), H&Y score (two-sample *t*-tests, *p* = 0.583), or UPDRS score (two-sample *t*-test, *p* = 0.490). Compared with familial PD groups, sporadic PD patients scored higher in HAMD (two-sample *t*-tests, *p* = 0.046), which suggested sporadic patients showed more severe depression and anxiety.

**Table 1:** Clinical and demographic characteristics.

**Index**	**FPD (*n* = 31)**	**SPD (*n* = 36)**	***p*-value**
Age (Y)	53.1 (±9.97)	53.8 (±11.7)	0.899^*^
Gender (m/f)	(17/15)	(21/15)	0.899^*^
Duration of disease (Y)	5.72 (±4.00)	4.66 (±4.53)	0.411^*^
Score of UPDRS	50.1 (±26.4)	45.5 (±23.1)	0.490^*^
Disease stage (H&Y)	2.39 (±0.58)	2.27 (±0.94)	0.583^*^
MMSE	28.13 (±1.57)	25.57 (±5.6)	0.063^*^
HAMD	6.65 (±5.83)	11.18 (±8.6)	0.046^*^
Side initially affected, L/R	17/14	19/17	0.5^•^
L-Dopa dose (mg/d)	434 ± 242	333 ± 243	0.174^*^
Percentage of patients treated with pramipexole	11	8	0.221^•^
Percentage of patients treated with piribedil	5	7	0.743^•^

*Data are expressed as mean ± SD. FPD-Familial Parkinson's Disease; SPD-Sporadic Parkinson's Disease; NC-Normal Control; H&Y, Hoehn and Yahr; UPDRS, Unified Parkinson's Disease Rating Scale; MMSE, Mini-Mental Status; HAMD, Hamilton Depression Scale*.

* Two-sample t-test;

• *Pearson χ^2^-test*.

### Altered network organization of the functional connectome

**Figure 1:**
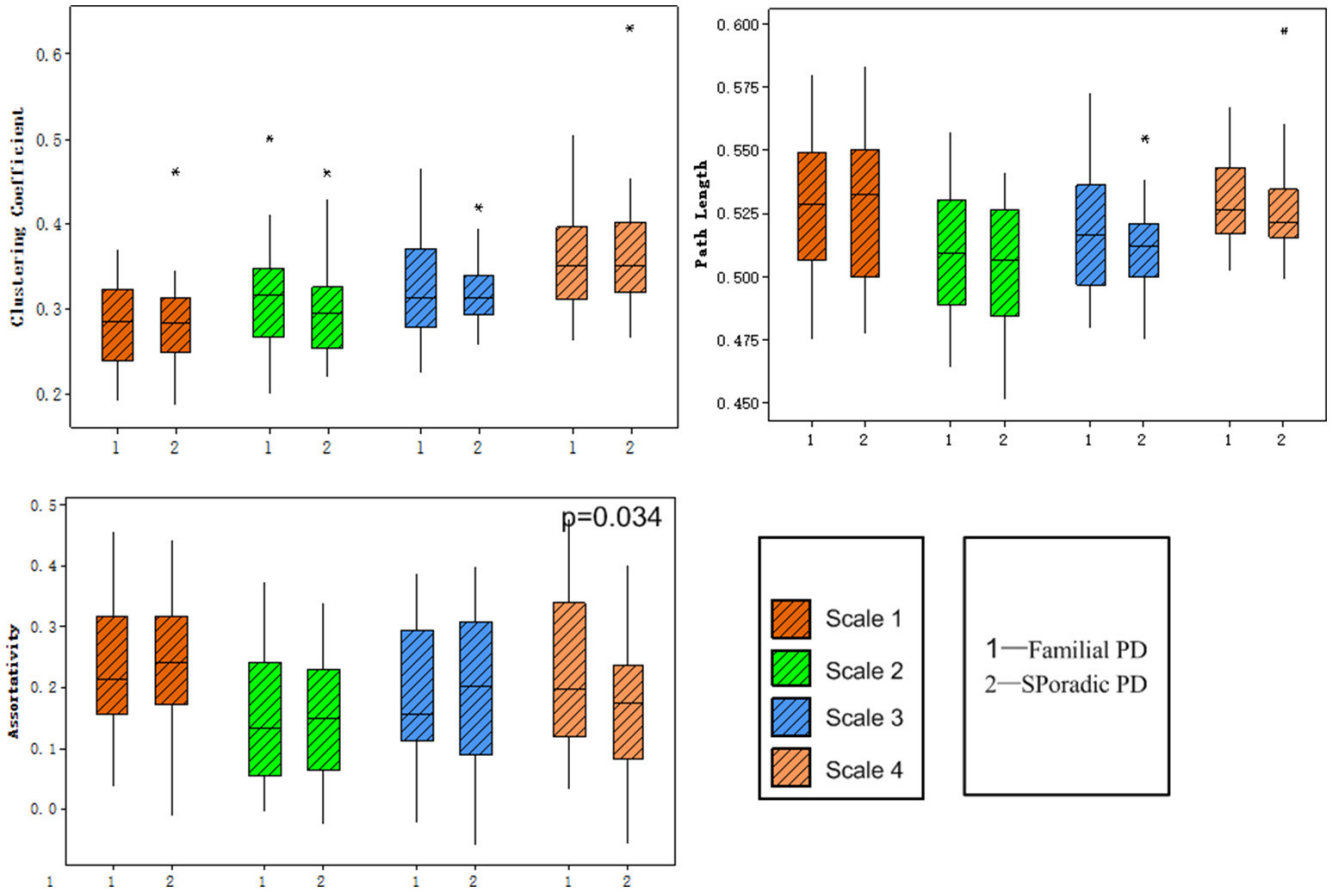
Group comparisons of global topological metrics across four wavelet scales. These metrics include clustering coefficient **(upper left)**, characteristic path length **(upper right)** and assortativity **(lower left)**.

**Figure 2:**
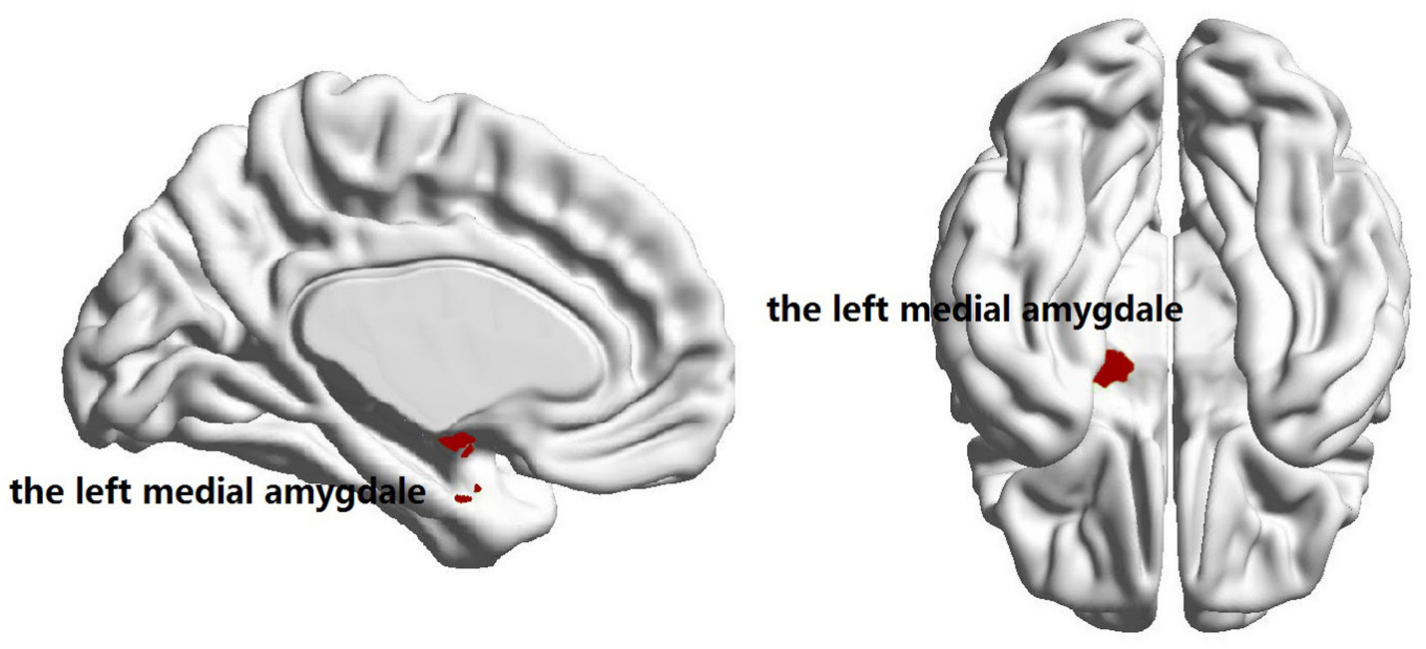
Brain regions showing the decreased nodal degree in familial PD group compared with sporadic PD group (*p* = 3 × 10^−5^) on the fourth wavelet scale.

No significant group difference was found in terms of the total numbers of supra-threshold links or the mean wavelet coefficients in the wavelet correlation matrices on any scales. Both the familial PD group and the sporadic PD group exhibited typical property of small-world topology with γ > 1 and λ≈1. However, the familial PD group exhibited a significant increase in assortativity (*p* = 0.034) on the fourth wavelet scale (0.016 −0.031 Hz) (Figure 1). A decreased nodal strength was observed in the left amygdala (medial amygdala) on the fourth scale (FDR-corrected *p* < 0.05, see Figure 2).

**Figure 3:**
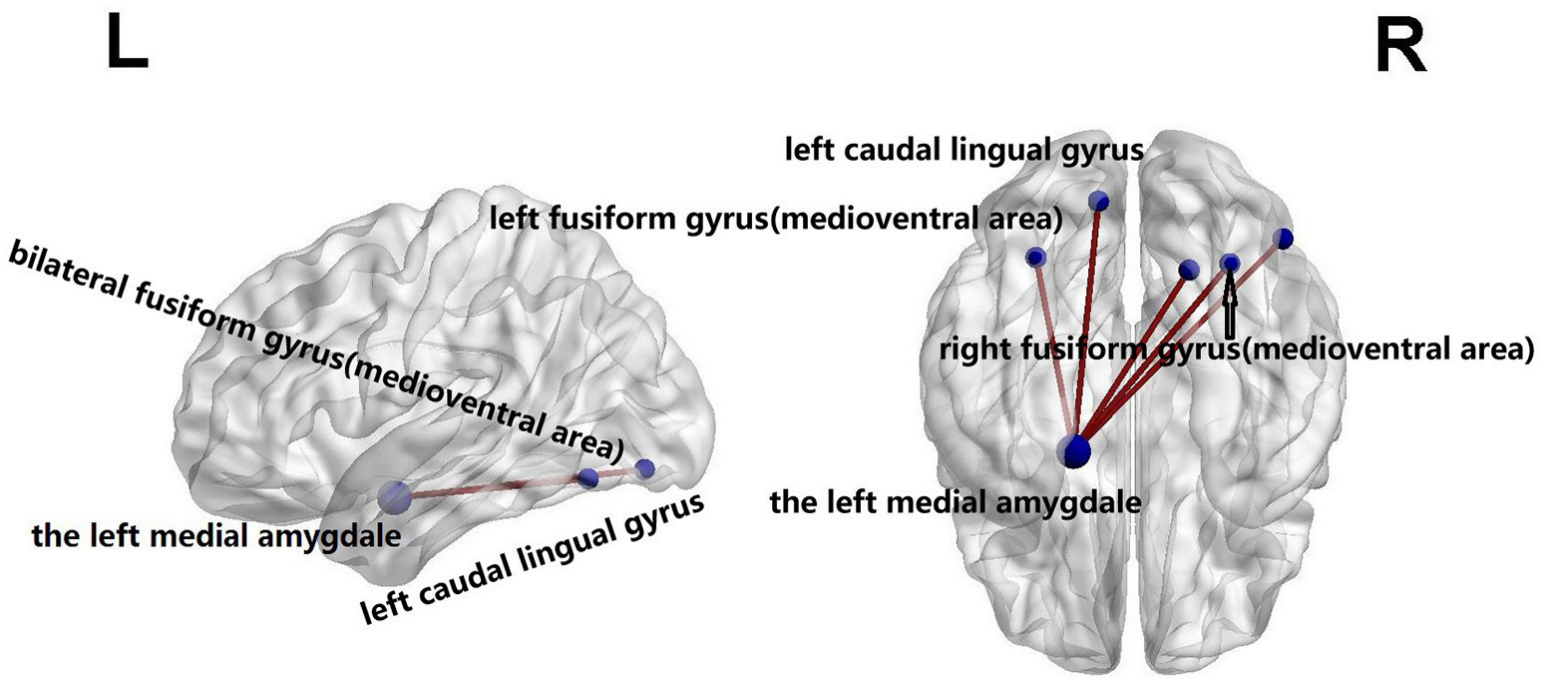
Compared with sporadic PD, familial PD group showed a decreased functional networks strength according to the network-based statistical (NBS) analysis method, under a liberal primary threshold of (*p* < 1 × 10^−3^). The line represents the functional connection.

Using the NBS analysis, five decreased functional connections were revealed in the familial PD group on the fourth scale, under the primary threshold (*p* < 1 × 10^−3^). Moreover, as shown in Figure 3, the left amygdala (medial amygdala) was found as the center of those functional connections with decreased strength, which connected to regions responsible for retrieval of motion information (Ueno et al., 2009) including the bilateral fusiform gyrus (medioventral area), left caudal lingual gyrus, right rostral lingual gyrus, and the right V5/MT+ area.

### Reproducibility of the results

As shown in Table 2, only on the fourth wavelet scale, significant group difference in network properties was found between the familial PD and sporadic PD group. Compared with sporadic PD, familial PD group showed significantly higher assortativity of weighted networks and the decreased nodal strength (FDR-corrected *p* < 0.05) in the left amygdala (medial amygdala).

**Table 2:** Comparisons of the global network metrics among the sporadic PD and familial PD on wavelet scale four.

		**L_p_**	**C_p_**	**λ**	**γ**	**σ**	**Assortativities**
familial PD	Case 0	0.530 ± 0.017	0.357 ±0.061	1.004 ± 0.006	1.247 0.184	1.241 0.177	0.216 ± 0.114
	Case 1	0.716 ± 0.085	0.249 ± 0.096	0.716 ± 0.085	1.0261 ± 0.0610	1.458 ± 0.211	0.247 ± 0.132
	Case 2	0.529 ± 0.017	0.358 ± 0.060	1.004 ± 0.007	1.240 ± 0.181	1.234 ± 0.173	0.217 ± 0.116
sporadic PD	Case 0	0.527 ± 0.019	0.363 ± 0.068	1.004 ± 0.005	1.263 ± 0.201	1.258 ± 0.194	0.169 ± 0.114
	Case 1	0.771± 0.313	0.267 ± 0.115	0.771 ± 0.313	1.434 ± 0.276	1.434 ± 0.276	0.195 ± 0.126
	Case 2	0.526 ± 0.018	0.365 ± 0.068	1.004 ± 0.005	1.257 ± 0.205	1.252 ± 0.198	0.170 ± 0.109
*p*-value	Case 0	0.287	0.261	0.433	0.257	0.277	0.034^*^
	Case 1	0.261	0.254	0.433	0.267	0.361	0.039^*^
	Case 2	0.322	0.336	0.433	0.257	0.359	0.042^*^

Data are expressed as mean ± SD (

* p < 0.05)

*C_p_: clustering coefficient; L_p_: characteristic path length of a network; γ: normalized clustering coefficient; λ: normalized characteristic path length; σ: small-worldness index; Case 0, negative connectivities were set to zero and “scrubbing” method was not applied; Case 1, negative connectivities were kept; Case 2, the “scrubbing” method was applied to alleviate the effect of head motion*.

On the fourth wavelet scale, compared with healthy control, sporadic PD patients also showed decreased assortativity. However, no significant difference in network properties was found between familial PD group and healthy controls (see Supplementary Material).

## Discussion

In this work, we explored the differences of topological organizations of the functional brain networks of subjects with sporadic and familial PD, and shed some light on the pathological mechanism impaired by this neurodegenerative disease. Compared to familial PD patients, altered functional connectome among sporadic PD group was found to predominant in the frequency band between 0.016 and 0.031 Hz, which is within the frequency range (0.01–0.073 Hz) of gray matter-related oscillations (Zuo et al., 2010). The alterations in functional organization between two PD groups can be characterized as the following: (i) a decrease in assortativity, (ii) increased nodal degree in the left medial amygdala, and (iii) changed functional connectivity between the left medial amygdala and brain regions related to the retrieval of motion information.

It has been shown by a previous study that neuronal fluctuations are linearly distributed on the natural logarithmic scale (Penttonen and Buzsáki, 2003). Despite the exact underlying mechanism being poorly understood, aberrant topological architectures in some specific frequency bands reflected the change of brain complexity depending on the type of the information being processed (Hanslmayr et al., 2012; Fontolan et al., 2014). In terms of PD, it has been found that the abnormal amplitude of low-frequency fluctuations (ALFF) is present in the frequency interval of 0.01–0.027 Hz (Zhang et al., 2013). Recent studies have also highlighted the significance of this frequency band in other brain diseases. For example, increases of brain functional network efficiency caused by nicotine were found in the frequency band between 0.01 and 0.03 Hz, which suggested cholinergic effects on network topology (Wylie et al., 2012). Antisocial personality disorder patients exhibited altered topological configuration of the functional connectome in the frequency interval of 0.016–0.031 Hz (Tang et al., 2016). These previous findings along with our present results highlight the clinical significance of spontaneous neural fluctuations between 0.016 and 0.031 Hz.

In the further analyse, the assortativity metric within the abovementioned frequency range, which is a measure of brain network resilience (Newman, 2002, 2003), differentiated the sporadic PD group from familial PD group. Networks with positive assortativity are found to be more resilient with more mutually interconnected high-degree nodes. In healthy brain organization, higher assortativity signals higher resilience to random error (Achard et al., 2006). The decreased resilience observed in some pathological conditions is interpreted as a loss of network functionality (Liu et al., 2011; Haneef and Chiang, 2014). Furthermore, altered nodal strength was only observed in the left medial amygdala, which suggested changed blood flow and metabolism in that brain region (Liang et al., 2013). The amygdala, which is linked to both cortical and subcortical networks, exhibits significant pathological changes in PD, as Braak et al. (2006) demonstrated that the amygdala is involved in different stage of PD development. The amygdala, is involved in processing emotional stimuli and forming emotional memories (Phelps, 2006). The right amygdala is important for fear processing, and the left amygdala is responsible for processing sadness and anxiety (Kienast et al., 2008). Depressed PD patients showed increased left amygdala connectivity (Hu et al., 2015), which is coincident with our found that sporadic PD patients might experience more severe depression and anxiety (see HAMD score in Table 1).

It is also worth mentioning that the left amygdala (medial amygdala) was found to be the hub of altered connections. Those changed connections were between the left amygdala and regions related to the retrieval of motion information, e.g., the bilateral fusiform gyrus (medioventral area), left caudal lingual gyrus, right rostral lingual gyrus, and right V5/MT+ area (Ueno et al., 2009). The medial fusiform gyrus may play a vital role in the integration of multiple stimuli (Zhang et al., 2016), and the lingual gyrus is involved in the analysis of logical conditions (i.e., the logical order of events) (Fusar-Poli et al., 2009), whereas V5/MT+ area retrieves motion information (Rosen, 2003; Ueno et al., 2009; Tang et al., 2017). Those regions with significantly changed connectivities are in line with findings of previous studies. Namely, Herrera et al. (2012) showed PD patients with a deficit in the retrieval of motion-related semantic content, and Dujardin et al. (2001) found that memory and executive function were more impaired in patients with sporadic PD than familial PD patients. In our study, compared with the healthy controls, sporadic PD group had lower MMSE score. Besides genetic mutations as a major contributor to both forms of PD, sporadic PD patients are also vulnerable to risk factors such as age, drug abuse, and gene-environment interactions (Hirsch et al., 2013; Perez, 2015). The non-motor symptoms occur in both PD groups, but appear to be more benign in familial PD than in sporadic PD (Alcalay et al., 2011; Yokoyama et al., 2011; Ferrer et al., 2012). Thus all results consistently suggest the sporadic PD patients might be more impaired in terms of cognition and it potentially reflects the nondopaminergic dysfunction in sporadic PD (Dujardin et al., 2001). Furthermore, the relative increases in functional connections in sporadic PD compared with familial PD group might provide evidence for the presence of compensatory neural mechanisms (Huang et al., 2007; Niethammer and Eidelberg, 2012) in sporadic PD patients relative to familial PD patients.

To our knowledge, our work is the first to explore frequency-dependent functional topological alterations between sporadic PD and familial PD. The difference of connectomes between sporadic PD and familial PD was identified and its pathological indication was discussed. Our findings not only provide novel insight into the neuropathological mechanisms of sporadic PD and familial PD but also highlight the potential clinical significance of frequency-dependent functional topology associated with different forms of PD.

## Author contributions

YT, BT, and JG contributed to the conception and design of the study. LM, CW, ZL, and WL conducted clinical assessments and MRI data collection. YT, XX, and CW performed data analysis. YT, JG, and HX wrote and critically revised the manuscript. JG takes full responsibility for the work and decision making.

### Conflict of interest statement

The authors declare that the research was conducted in the absence of any commercial or financial relationships that could be construed as a potential conflict of interest.

## References

[B1] Achard S. Salvador R. Whitcher B. Suckling J. Bullmore E. (2006). A resilient, low-frequency, small-world human brain functional network with highly connected association cortical hubs. J. Neurosci. 26, 63–72. 10.1523/JNEUROSCI.3874-05.2006 16399673 PMC6674299

[B2] Alcalay R. N. Siderowf A. Ottman R. Caccappolo E. Mejiasantana H. Tang M. X.. (2011). Olfaction in Parkin heterozygotes and compound heterozygotes: the CORE-PD study. Neurology 76, 319. 10.1212/WNL.0b013e31820882aa 21205674 PMC3034420

[B3] Baria A. T. Baliki M. N. Parrish T. Apkarian A. V. (2011). Anatomical and functional assemblies of brain BOLD oscillations. J. Neurosci. 31, 7910–7919. 10.1523/JNEUROSCI.1296-11.2011 21613505 PMC3114444

[B4] Barzegaran E. Joudaki A. Jalili M. Rossetti A. O. Frackowiak R. S. Knyazeva M. G. (2012). Properties of functional brain networks correlate frequency of psychogenic non-epileptic seizures. Front. Hum. Neurosci. 6:335. 10.3389/fnhum.2012.00335 23267325 PMC3526831

[B5] Braak H. Bohl J. R. Müller C. M. Rüb U. de Vos R. A. Del Tredici K. (2006). Stanley fahn lecture 2005: the staging procedure for the inclusion body pathology associated with sporadic Parkinson's disease reconsidered. Mov. Disord. 21, 2042–2051. 10.1002/mds.21065 17078043

[B6] Buzsáki G. Draguhn A. (2004). Neuronal oscillations in cortical networks. Science 304, 1926–1929. 10.1126/science.1099745 15218136

[B7] Chai C. Lim K.-L. (2013). Genetic insights into sporadic Parkinson's disease pathogenesis. Curr. Genomics 14, 486–501. 10.2174/1389202914666131210195808 24532982 PMC3924245

[B8] Chao-Gan Y. Yu-Feng Z. (2010). DPARSF: a MATLAB toolbox for “pipeline” data analysis of resting-state fMRI. Front. Syst. Neurosci. 4:13. 10.3389/fnsys.2010.00013 20577591 PMC2889691

[B9] Ciceri F. Rotllant D. Maes T. (2017). Understanding epigenetic alterations in Alzheimer's and Parkinson's disease: towards targeted biomarkers and therapies. Curr. Pharm. Des. 23, 839–857. 10.2174/1381612823666170124121140 28120717

[B10] Desjardins A. E. Kiehl K. A. Liddle P. F. (2001). Removal of confounding effects of global signal in functional MRI analyses. Neuroimage 13, 751–758. 10.1006/nimg.2000.0719 11305902

[B11] Di Martino A. Ross K. Uddin L. Q. Sklar A. B. Castellanos F. X. Milham M. P. (2009). Functional brain correlates of social and nonsocial processes in autism spectrum disorders: an activation likelihood estimation meta-analysis. Biol. Psychiatry 65, 63–74. 10.1016/j.biopsych.2008.09.022 18996505 PMC2993772

[B12] Dujardin K. Defebvre L. Grunberg C. Becquet E. Destée A. (2001). Memory and executive function in sporadic and familial Parkinson's disease. Brain 124, 389–398. 10.1093/brain/124.2.389 11157566

[B13] Fahn S. Elton R. L. (1987). Unified Parkinson's disease rating scale in Recent Developments in Parkinson's Disease, eds Fahn S. Marsden C. D. Calne D. Goldstein M. (Florham Park, NJ: Macmillan Health Care Information), 153–163.

[B14] Fan L. Li H. Zhuo J. Zhang Y. Wang J. Chen L.. (2016). The human brainnetome atlas: a new brain atlas based on connectional architecture. Cereb. Cortex 26, 3508–3526. 10.1093/cercor/bhw157 27230218 PMC4961028

[B15] Fang J. Chen H. Cao Z. Jiang Y. Ma L. Ma H.. (2017). Impaired brain network architecture in newly diagnosed Parkinson's disease based on graph theoretical analysis. Neurosci. Lett. 657, 151–158. 10.1016/j.neulet.2017.08.002 28789983

[B16] Ferrer I. Lópezgonzalez I. Carmona M. Dalfó E. Pujol A. Martínez A. (2012). Neurochemistry and the non-motor aspects of PD. Neurobiol. Dis. 46, 508. 10.1016/j.nbd.2011.10.019 22737710

[B17] Folstein M. F. Folstein S. E. McHugh P. R. (1975). “Mini-mental state”: a practical method for grading the cognitive state of patients for the clinician. J. Psychiatr. Res. 12, 189–198. 10.1016/0022-3956(75)90026-6 1202204

[B18] Fontolan L. Morillon B. Liegeois-Chauvel C. Giraud A.-L. (2014). The contribution of frequency-specific activity to hierarchical information processing in the human auditory cortex. Nat. Commun. 5:4694. 10.1038/ncomms5694 25178489 PMC4164774

[B19] Fox M. D. Raichle M. E. (2007). Spontaneous fluctuations in brain activity observed with functional magnetic resonance imaging. Nat. Rev. Neurosci. 8, 700–711. 10.1038/nrn2201 17704812

[B20] Fox M. D. Zhang D. Snyder A. Z. Raichle M. E. (2009). The global signal and observed anticorrelated resting state brain networks. J. Neurophysiol. 101, 3270–3283. 10.1152/jn.90777.2008 19339462 PMC2694109

[B21] Fusar-Poli P. Placentino A. Carletti F. Landi P. Allen P. Surguladze S.. (2009). Functional atlas of emotional faces processing: a voxel-based meta-analysis of 105 functional magnetic resonance imaging studies. J. Psychiatry Neurosci. 34, 418–432. 19949718 PMC2783433

[B22] Goetz C. G. (2003). The Unified Parkinson's Disease Rating Scale (UPDRS): status and recommendations. Mov. Disord. 18, 738–750. 10.1002/mds.10473 12815652

[B23] Göttlich M. Münte T. F. Heldmann M. Kasten M. Hagenah J. Krämer U. M. (2013). Altered resting state brain networks in Parkinson's disease. PLoS ONE 8:e77336. 10.1371/journal.pone.0077336 24204812 PMC3810472

[B24] Guo J.-F. Xiao B. Liao B. Zhang X.-W. Nie L.-L. Zhang Y. H.. (2008). Mutation analysis of Parkin, PINK1, DJ-1 and ATP13A2 genes in Chinese patients with autosomal recessive early-onset parkinsonism. Mov. Disord. 23, 2074–2079. 10.1002/mds.22156 18785233

[B25] Hacker C. Perlmutter J. Criswell S. Ances B. Snyder A. (2012). Resting state functional connectivity of the striatum in Parkinson's disease. Brain 35(Pt 12), 3699–3711. 10.1093/brain/aws281 PMC3525055 23195207

[B26] Hamilton M. (1960). A rating scale for depression. J. Neurol. Neurosurg. Psychiatry 23:56. 10.1136/jnnp.23.1.56 14399272 PMC495331

[B27] Haneef Z. Chiang S. (2014). Clinical correlates of graph theory findings in temporal lobe epilepsy. Seizure 23:809. 10.1016/j.seizure.2014.07.004 25127370 PMC4281255

[B28] Hanslmayr S. Staudigl T. Fellner M.-C. (2012). Oscillatory power decreases and long-term memory: the information via desynchronization hypothesis. Front. Hum. Neurosci. 6:74. 10.3389/fnhum.2012.00074 22514527 PMC3322486

[B29] Hauser D. N. Hastings T. G. (2013). Mitochondrial dysfunction and oxidative stress in Parkinson's disease and monogenic parkinsonism. Neurobiol. Dis. 51:35. 10.1016/j.nbd.2012.10.011 23064436 PMC3565564

[B30] Herrera E. Rodríguezferreiro J. Cuetos F. (2012). The effect of motion content in action naming by Parkinson's disease patients. Cortex 48, 900–904. 10.1016/j.cortex.2010.12.007 21247557

[B31] Hirsch E. C. Jenner P. Przedborski S. (2013). Pathogenesis of Parkinson's disease. Mov. Disord. 28, 24–30. 10.1002/mds.25032 22927094

[B32] Hoehn M. M. Yahr M. D. (1967). Parkinsonism: onset, progression and mortality. Neurology 17, 427–442. 10.1212/WNL.17.5.427 6067254

[B33] Hu X. Song X. Yuan Y. Li E. Liu J. Liu W.. (2015). Abnormal functional connectivity of the amygdala is associated with depression in Parkinson's disease. Mov. Disord. 30, 238–244. 10.1002/mds.26087 25545969

[B34] Huang C. Mattis P. Tang C. Perrine K. Carbon M. Eidelberg D. (2007). Metabolic brain networks associated with cognitive function in Parkinson's disease. Neuroimage 34, 714. 10.1016/j.neuroimage.2006.09.003 17113310 PMC4456012

[B35] Hughes A. J. Daniel S. E. Kilford L. Lees A. J. (1992). Accuracy of clinical diagnosis of idiopathic Parkinson's disease: a clinico-pathological study of 100 cases. J. Neurol. Neurosurg. Psychiatry 55, 181–184. 10.1136/jnnp.55.3.181 1564476 PMC1014720

[B36] Hughes A. J. Daniel S. E. Lees A. J. (2001). Improved accuracy of clinical diagnosis of Lewy body Parkinson's disease. Neurology 57, 1497–1499. 10.1212/WNL.57.8.1497 11673599

[B37] Kienast T. Hariri A. R. Schlagenhauf F. Wrase J. Sterzer P. Buchholz H. G.. (2008). Dopamine in amygdala gates limbic processing of aversive stimuli in humans. Nat. Neurosci. 11, 1381–1382. 10.1038/nn.2222 18978778

[B38] Kriegeskorte N. Goebel R. Bandettini P. (2006). Information-based functional brain mapping. Proc. Natl. Acad. Sci. U.S.A. 103, 3863–3868. 10.1073/pnas.0600244103 16537458 PMC1383651

[B39] Li Y. Yao H. Lin P. Zheng L. Li C. Zhou B.. (2017). Frequency-dependent altered functional connections of default mode network in Alzheimer's disease. Front. Aging Neurosci. 9:259. 10.3389/fnagi.2017.00259 28824420 PMC5540901

[B40] Liang X. Zou Q. He Y. Yang Y. (2013). Coupling of functional connectivity and regional cerebral blood flow reveals a physiological basis for network hubs of the human brain. Proc. Natl. Acad. Sci. U.S.A. 110, 1929–1934. 10.1073/pnas.1214900110 23319644 PMC3562840

[B41] Liu J. Qin W. Nan J. Li J. Yuan K. Zhao L.. (2011). Gender-related differences in the dysfunctional resting networks of migraine suffers. PLoS ONE 6:e27049. 10.1371/journal.pone.0027049 22073251 PMC3206886

[B42] Luo C. Y. Guo X. Y. Song W. Chen Q. Cao B. Yang J.. (2015). Functional connectome assessed using graph theory in drug-naive Parkinson's disease. J. Neurol. 262, 1557–1567. 10.1007/s00415-015-7750-3 25929663

[B43] Lynall M.-E. Bassett D. S. Kerwin R. McKenna P. J. Kitzbichler M. Muller U.. (2010). Functional connectivity and brain networks in schizophrenia. J. Neurosci. 30, 9477–9487. 10.1523/JNEUROSCI.0333-10.2010 20631176 PMC2914251

[B44] Maraganore D. M. Harding A. E. Marsden C. D. (1991). A clinical and genetic study of familial Parkinson's disease. Mov. Disord. 6, 205–211. 10.1002/mds.870060303 1922124

[B45] Maxim V. Sendur L. Fadili J. Suckling J. Gould R. Howard R.. (2005). Fractional Gaussian noise, functional MRI and Alzheimer's disease. Neuroimage 25, 141–158. 10.1016/j.neuroimage.2004.10.044 15734351

[B46] Murphy K. Birn R. M. Handwerker D. A. Jones T. B. Bandettini P. A. (2009). The impact of global signal regression on resting state correlations: are anti-correlated networks introduced? Neuroimage 44, 893–905. 10.1016/j.neuroimage.2008.09.036 18976716 PMC2750906

[B47] Newman M. E. (2002). Assortative mixing in networks. Phys. Rev. Lett. 89, 111–118. 10.1103/PhysRevLett.89.208701 12443515

[B48] Newman M. E. (2003). Mixing patterns in networks. Phys. Rev. E Stat. Nonlin. Soft Matter Phys. 67(2 Pt 2), 241–251. 10.1103/PhysRevE.67.026126 12636767

[B49] Niethammer M. Eidelberg D. (2012). Metabolic brain networks in translational neurology: concepts and applications. Ann. Neurol. 72:635. 10.1002/ana.23631 22941893 PMC4564117

[B50] Palop J. J. Chin J. Mucke L. (2006). A network dysfunction perspective on neurodegenerative diseases. Nature 443:768. 10.1038/nature05289 17051202

[B51] Parkinson J. (2002). An Essay on the Shaking Palsy. Sherwood, OR: Neeley & Jones.

[B52] Penttonen M. Buzsáki G. (2003). Natural logarithmic relationship between brain oscillators. Thalamus Relat. Syst. 2, 145–152. 10.1017/S1472928803000074

[B53] Percival D. B. Walden A. T. (2006). Wavelet Methods for Time Series Analysis. Cambridge, UK: Cambridge University Press.

[B54] Perez X. A. (2015). Preclinical evidence for a role of the nicotinic cholinergic system in Parkinson's disease. Neuropsychol. Rev. 25, 371–383. 10.1007/s11065-015-9303-z 26553323

[B55] Phelps E. A. (2006). Emotion and cognition: insights from studies of the human amygdala. Annu. Rev. Psychol. 57, 27–53. 10.1146/annurev.psych.56.091103.070234 16318588

[B56] Power J. D. Barnes K. A. Snyder A. Z. Schlaggar B. L. Petersen S. E. (2012). Spurious but systematic correlations in functional connectivity MRI networks arise from subject motion. Neuroimage 59, 2142–2154. 10.1016/j.neuroimage.2011.10.018 22019881 PMC3254728

[B57] Rosen D. (2003). Amygdala abnormality and its role in autistic socio-emotional impairment: a proposed study of somatic intervention among macaque monkeys. Concept 27.

[B58] Rubinov M. Sporns O. (2010). Complex network measures of brain connectivity: uses and interpretations. Neuroimage 52, 1059–1069. 10.1016/j.neuroimage.2009.10.003 19819337

[B59] Sanz-Arigita E. J. Schoonheim M. M. Damoiseaux J. S. Rombouts S. A. Maris E. Barkhof F.. (2010). Loss of 'small-world'networks in Alzheimer's disease: graph analysis of FMRI resting-state functional connectivity. PloS ONE 5:e13788. 10.1371/journal.pone.0013788 21072180 PMC2967467

[B60] Skidmore F. Korenkevych D. Liu Y. He G. Bullmore E. Pardalos P. M. (2011). Connectivity brain networks based on wavelet correlation analysis in Parkinson fMRI data. Neurosci. Lett. 499, 47–51. 10.1016/j.neulet.2011.05.030 21624430

[B61] Sporns O. Tononi G. Kötter R. (2005). The human connectome: a structural description of the human brain. PLoS Comput. Biol. 1:e42. 10.1371/journal.pcbi.0010042 16201007 PMC1239902

[B62] Supekar K. Menon V. Rubin D. Musen M. Greicius M. D. (2008). Network analysis of intrinsic functional brain connectivity in alzheimer's disease. PLoS Comput. Biol. 4:e1000100 10.1371/journal.pcbi.1000100 18584043 PMC2435273

[B63] Tang Y. Long J. Wang W. Liao J. Xie H. Zhao G.. (2016). Aberrant functional brain connectome in people with antisocial personality disorder. Sci. Rep. 6:26209. 10.1038/srep26209 27257047 PMC4891727

[B64] Tang Y. Meng L. Wan C.-M. Liu Z.-H. Liao W.-H. Yan X.-X.. (2017). Identifying the presence of Parkinson's disease using low-frequency fluctuations in BOLD signals. Neurosci. Lett. 645, 1–6. 10.1016/j.neulet.2017.02.056 28249785

[B65] Tessitore A. Amboni M. Esposito F. Russo A. Picillo M. Marcuccio L.. (2012). Resting-state brain connectivity in patients with Parkinson's disease and freezing of gait. Parkinsonism Relat. Disord. 18, 781–787. 10.1016/j.parkreldis.2012.03.018 22510204

[B66] Thompson P. M. Cannon T. D. Narr K. L. van Erp T. Poutanen V. P. Huttunen M.. (2001). Genetic influences on brain structure. Nat. Neurosci. 4:1253. 10.1038/nn758 11694885

[B67] Ueno A. Abe N. Suzuki M. Shigemune Y. Hirayama K. Mori E.. (2009). Reactivation of medial temporal lobe and human V5/MT+ during the retrieval of motion information: a PET study. Brain Res. 1285, 127–134. 10.1016/j.brainres.2009.06.025 19527693

[B68] Wang J. Zuo X. Dai Z. Xia M. Zhao Z. Zhao X.. (2013). Disrupted functional brain connectome in individuals at risk for Alzheimer's disease. Biol. Psychiatry 73, 472–481. 10.1016/j.biopsych.2012.03.026 22537793

[B69] Wang J. H. Zuo X. N. Gohel S. Milham M. P. Biswal B. B. He Y. (2011). Graph theoretical analysis of functional brain networks: test-retest evaluation on short- and long-term resting-state functional MRI data. PLoS ONE 6:e21976. 10.1371/journal.pone.0021976 21818285 PMC3139595

[B70] Wylie K. P. Rojas D. C. Tanabe J. Martin L. F. Tregellas J. R. (2012). Nicotine increases brain functional network efficiency. Neuroimage 63, 73–80. 10.1016/j.neuroimage.2012.06.079 22796985 PMC3429645

[B71] Yokoyama H. Uchida H. Kuroiwa H. Kasahara J. Araki T. (2011). Role of glial cells in neurotoxin-induced animal models of Parkinson's disease. Neurol. Sci. 32, 1–7. 10.1007/s10072-010-0424-0 21107876

[B72] Yu R. Chien Y. L. Wang H. L. S. Liu C. M. Liu C. C. Hwang T. J.. (2014). Frequency-specific alternations in the amplitude of low-frequency fluctuations in schizophrenia. Hum. Brain Mapp. 35, 627–637. 10.1002/hbm.22203 23125131 PMC6869729

[B73] Zalesky A. Fornito A. Bullmore E. T. (2010). Network-based statistic: identifying differences in brain networks. Neuroimage 53, 1197–1207. 10.1016/j.neuroimage.2010.06.041 20600983

[B74] Zhang W. Wang J. Fan L. Zhang Y. Fox P. T. Eickhoff S. B.. (2016). Functional organization of the fusiform gyrus revealed with connectivity profiles. Hum. Brain Mapp. 37, 3003–3016. 10.1002/hbm.23222 27132874 PMC6867330

[B75] Zhang D. Wang J. Liu X. Chen J. Liu B. (2015). aberrant brain network efficiency in Parkinson's disease patients with tremor: a multi-modality study. Front. Aging Neurosci. 7:169. 10.3389/fnagi.2015.00169 26379547 PMC4553412

[B76] Zhang J. Wei L. Hu X. Zhang Y. Zhou D. Li C. (2013). Specific frequency band of amplitude low-frequency fluctuation predicts Parkinson's disease. Behav. Brain Res. 252, 18–23. 10.1016/j.bbr.2013.05.039 23727173

[B77] Zuo X.-N. Di Martino A. Kelly C. Shehzad Z. E. Gee D. G. Klein D. F.. (2010). The oscillating brain: complex and reliable. Neuroimage 49, 1432–1445. 10.1016/j.neuroimage.2009.09.037 19782143 PMC2856476

[B78] Zuo X. N. Ehmke R. Mennes M. Imperati D. Castellanos F. X. Sporns O.. (2012). Network centrality in the human functional connectome. Cereb. Cortex 22, 1862–1875. 10.1093/cercor/bhr269 21968567

