# Supplementary material for: Altered Functional Brain Connectomes between Sporadic and Familial Parkinson's Patients
Source: Front Neuroanat. 2017 Nov 6;11:99. doi: 10.3389/fnana.2017.00099 (PMC5681528; doi:10.3389/fnana.2017.00099)
Supplement: Supplementary file 1 [file DataSheet1.DOCX]

**Altered functional brain connectomes between sporadic and familial Parkinson's patients**

**Yan Tang MD, MS ^1,4^, Xue Xiao BS^5^ , Hua Xie MS ^8^, Chang-min Wan MB ^1^, Li Meng MD ^3^, Zhen-hua Liu MD ^1^, Wei-hua Liao MD ^3^, Bei-sha Tang MD^1,2,5,6,7^, Ji-feng Guo MD^1,2^***

**Supplementary Materials**

# Material and methods

**Subjects**

A group of 38 normal control (NC) subjects was chosen to match the two groups of patients with Parkinson's disease (sporadic PD and familial PD) in terms of age and sex. They had no parkinsonian symptoms and had never received neuroleptic treatment. The healthy controls have no family of PD.

# Results

**Demographic and Psychometric Information**

As displayed in Table S1, there were no age or sex differences among the three groups (see Table S1). The normal controls showed no difference compared to sporadic PD (SPD) and familial PD (FPD) patients in terms of age using *t*-test (SPD vs NC *p* = 0.799; FPD vs. NC *p* = 0.838) and gender (Chi-square test FPD vs. NC *p* = 0.745; SPD vs NC *p* = 0.834). Compared with NC, sporadic PD patients scored lower in MMSE (*p* = 0.018) and no significant group difference was found between familial PD and NC in this regard *(p* = 0.063).

**Altered Network Organization of the Functional Connectome**

Clustering coefficients, characteristic path length, global efficiency, and assortativity were evaluated on the fourth wavelet scale of the wavelet correlation matrices. Both PD groups and NC exhibited typical property of small-world topology with γ > 1 and $\lambda\approx1$. Results of pairwise comparisons were reported in Figure S1. On the fourth wavelet scale, compared with NC, sporadic PD showed decreased assortativity. However, no significant difference in terms of other graphical measures (i.e. clustering coefficients, characteristic path length, and global efficiency) were found group between PD groups and NC, which could be due to compensation of alternative pathways.

Table S1. Clinical and demographic characteristics.

Data are expressed as mean ± SD. FPD- Familial Parkinson’s Disease; SPD- Sporadic Parkinson’s Disease; NC- Normal Control; H & Y–Hoehn and Yahr; UPDRS–Unified Parkinson’s Disease Rating Scale; MMSE–Mini-Mental Status; HAMD- Hamilton Depression Scale. *Two-sample t-test; #Chi-square test; *●*Pearson χ^2^-test

| Index | FPD (n=31) | SPD (n=36) | FPD vs SPD p-value | NC (n=38) | FPD vs NC p-value | SPD vs NC p-value |
| --- | --- | --- | --- | --- | --- | --- |
| Age (Y) | 53.1(±9.97) | 53.8(±11.7) | 0.899^*^ | 53.4((±5.97) | 0.838^*^ | 0.799^*^ |
| Gender (m/f) | (17/15) | (21/15) | 0.892^#^ | (21/17) | 0.745^#^ | 0.834^#^ |
| Duration of disease (Y) | 5.72(±4.00) | 4.66(±4.53) | 0.411^*^ | NA | NA | NA |
| Score of UPDRS | 50.1(±26.4) | 45.5(±23.1) | 0.490^*^ | NA | NA | NA |
| Disease stage (H&Y) | 2.39(±0.58) | 2.27(±0.94) | 0.583^*^ | NA | NA | NA |
| MMSE | 28.1(±1.57) | 25.57(±5.6) | 0.063^*^ | 28.171(±1.6) | 0.105^*^ | 0.018^*^ |
| HAMD | 6.65(±5.83) | 11.18(±8.6) | 0.046^*^ | NA | NA | NA |
| Side initially affected, L/R | 17/14 | 19/17 | 0.5^●^ | NA | NA | NA |
| L-Dopa dose (mg/d) | 434±242 | 333±243 | 0.174^*^ | NA | NA | NA |
| Percentage of patients treated with pramipexole | 11 | 8 | 0.221^●^ | NA | NA | NA |
| Percentage of patients treated with piribedil | 5 | 7 | 0.743^●^ | NA | NA | NA |


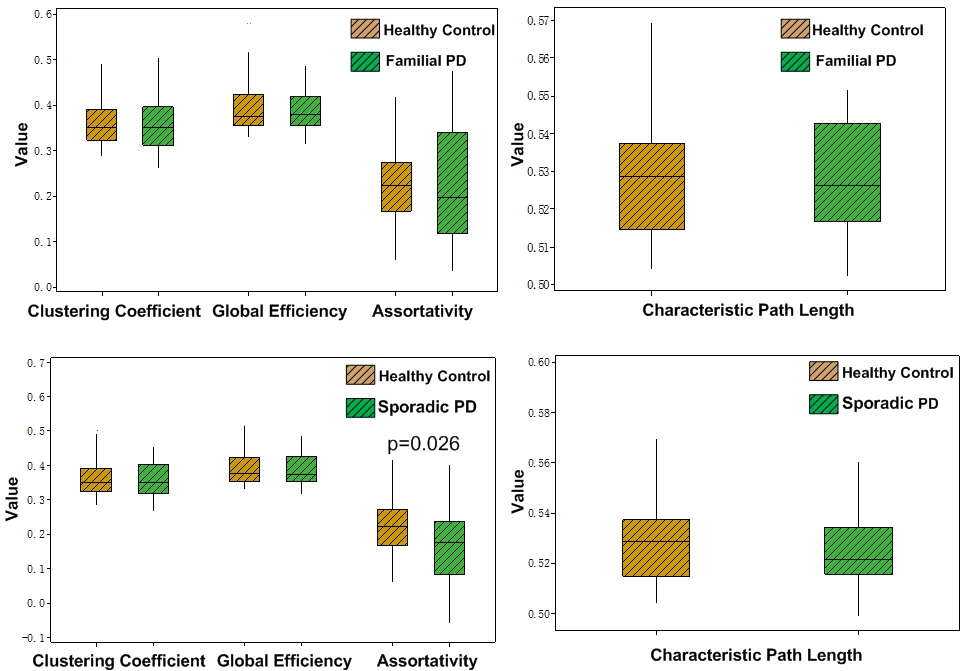


Figure S1 **Group comparisons of global topological metrics on the four wavelet scales. These metrics include clustering coefficient, global efficiency,** **characteristic path length and assortativity.**
